# Impact of Quenching Failure of Cy Dyes in Differential Gel Electrophoresis

**DOI:** 10.1371/journal.pone.0018098

**Published:** 2011-03-30

**Authors:** Weiqun Wang, Doreen Ackermann, Anna-Maria Mehlich, Simone König

**Affiliations:** Integrated Functional Genomics, Core Unit of the Interdisciplinary Center for Clinical Research, Medical Faculty, University of Münster, Münster, Germany; University of Pennsylvania, United States of America

## Abstract

**Background:**

Differential gel electrophoresis (DIGE) is a technology widely used for protein expression analysis. It is based on labelling with fluorescent Cy dyes. In comparative fluorescence gel electrophoresis experiments, however, unspecific labelling using *N*-hydroxy-succinimide-ester-based labelling protocols was recently detected. Cross-talk was observed due to failure of the quenching process. Here, the impact of this effect for DIGE experiments was investigated.

**Methodology/Principal Findings:**

Experiments to test quenching efficiency were performed in replicate using *Escherichia coli* lysate. Parameters such as the amount of dye and quencher were varied. Labelling and quenching were reversed in one experiment. Differences in protein spot volumes due to limited quenching were determined. For some spots twice the volume was detected underscoring the importance of proper control of silencing of active dye.

**Conclusions/Significance:**

It could be demonstrated that uncontrolled labelling increased protein spot volume, even doubling it in some cases. Moreover, proteins responded differently to the protocol. Such unpredictable and unspecific processes are not acceptable in protein regulation studies so that it is necessary to validate the correct amount of quencher for individual samples before the DIGE experiment is performed. Increase of the concentration of lysine, which is used as quencher, from 10 mM to 2500 mM, was sufficient to silence the dye. Alternatively, active dye molecules can be removed by filtration.

## Introduction

It was recently shown [1] that quenching of *N*-hydroxy-succinimide (NHS)-ester-coupled dyes in comparative fluorescence gel electrophoresis may require a major excess of quencher in order to avoid unspecific labelling. As much as 2.500-fold excess of reaction partners needs to be present to silence the activated dye molecules sufficiently. This is about 100-fold more quencher than is typically used in differential gel electrophoresis (DIGE) [2], a technique, which has been widely employed for protein expression analysis during the past decade [3]. DIGE is a well-designed experimental set-up which represents the state-of-the-art in gel-based regulation studies. The gel-to-gel variation has been reduced to a minimum applying several samples to one gel. This is possible due to labelling with charge- and mass-matched cyanine fluorescent dyes. An internal standard formed by all samples participating in the experiment is furthermore introduced. Software support for statistical data analysis has also been provided. DIGE experiments take 2-4 weeks, are cost-intensive and consume valuable sample material (biological replicates) so that it is important to critically evaluate the influence of the detected cross-talk.

When the principle of DIGE was first introduced by Ünlü *et al* in 1997 [4], 1,3-diamino-2-hydroxy-propane was used for quenching and, moreover, active dye molecules were physically removed from the solution containing the labelled proteins by overnight adsorption to SM-2 beads. In later work by other authors [5], lysine was introduced as quencher and the dye-removal step was omitted. Quencher was provided in excess (200 pmol dye, 50 µg protein, 10 nmol lysine) so that its was reasonable to assume complete silencing of active dye. This protocol provided the basis for the commercial DIGE system with the only difference that 400 pmol dye labeled 50 µg of protein. It has been evaluated and applied by many researchers including us for protein expression analysis. Ünlü and coworkers have published another protocol [6] using methylamine-HCl and HEPES for quenching, this time not removing the residual dye from the protein solution. We have not used this method and do therefore not discuss it below.

Unspecific labelling is expected to cause increased spot volumes in DIGE. This is due, first, to continued labelling with the dye assigned to the individual sample, because the labelling time cannot be controlled and, second, to unspecific labelling of the other two samples of the set when all three samples are pooled in preparation for isoelectric focussing (Figure S1). We have shown extensively before [1] that the recommended amount of the quencher lysine is not sufficient in labelling protocols as they are used in DIGE [2]; cross-talk of one proteome (sample 1, e.g. control) in the fluorescent gel image of the second proteome (sample 2, e.g. sample of interest) was routinely detected. Different amounts of dye, protein and quencher were tested. To elucidate the impact of limited quenching on expression data, comparative experiments using *Escherichia coli* cell lysate were performed in this work.

## Results and Discussion

**Figure 1:**
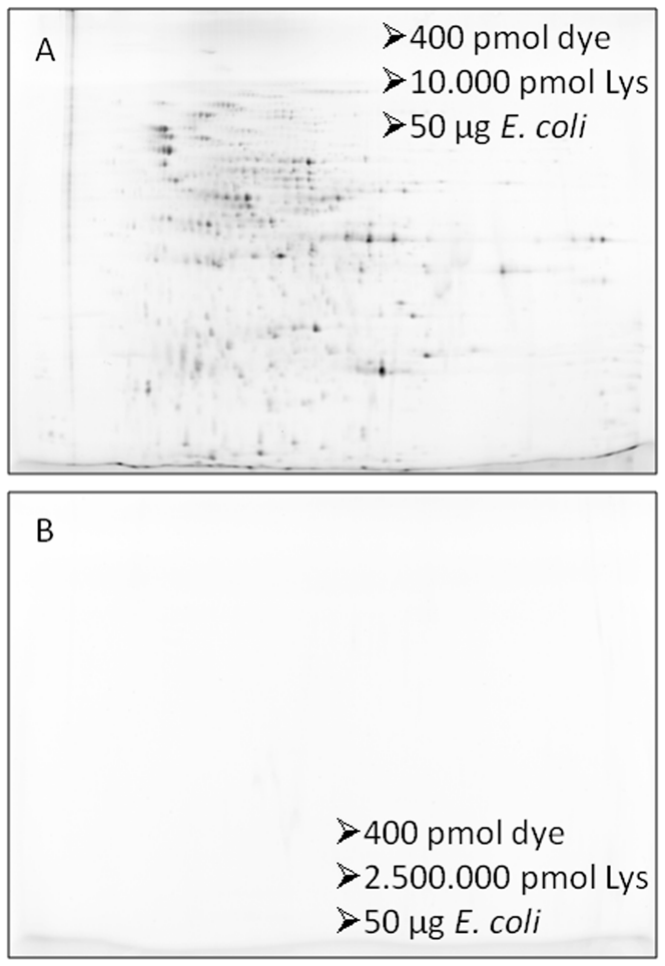
Reversal of labelling and quenching. Cy2 image of *E. coli* lysate (pH 3–10, molecular weight 10–150 kDa). Protein was added after quenching. **A**) Regular protocol [2]; 10 mM lysine. Protein spots are detected on the gel. **B**) 250 times more quencher silences the active dye (lysine to dye ratio 1∶6250 instead of 1∶25).

Building on the earlier experiments [1], we first reversed labelling and quenching. To that end, 400 pmol Cy2, Cy3, and Cy5, respectively, were quenched with 10.000 pmol lysine as advised [2] and then 50 µg protein were added (for experimental design and protocols see Text S1, Table S1 and Table S2). At this stage in the protocol the dye should be silenced; if it is not, unspecific labelling during sample pooling would occur in DIGE. As can be seen in Figure 1 for Cy2, the proteome was still visualized on the gel although the image should be blank; this was only achieved when 250 times as much lysine was added. That was the maximum amount of quencher which could be used under these conditions for solubility reasons. Similar results were observed for Cy3 and Cy5 (Figure S2). Intermediate concentrations of lysine were tested before [1]. Instead of increasing the amount of quencher one could, in principle, also use less dye. A tenth of the recommended dye amount was added (40 pmol) for protein labelling, but 10-fold more quencher (Figure S2). However, not unexpectedly, sensitivity was lowered and spots were lost. We did not test for further, intermediate, concentrations, because the recommended labelling protocol was a minimal labelling approach in the first place.

**Figure 2:**
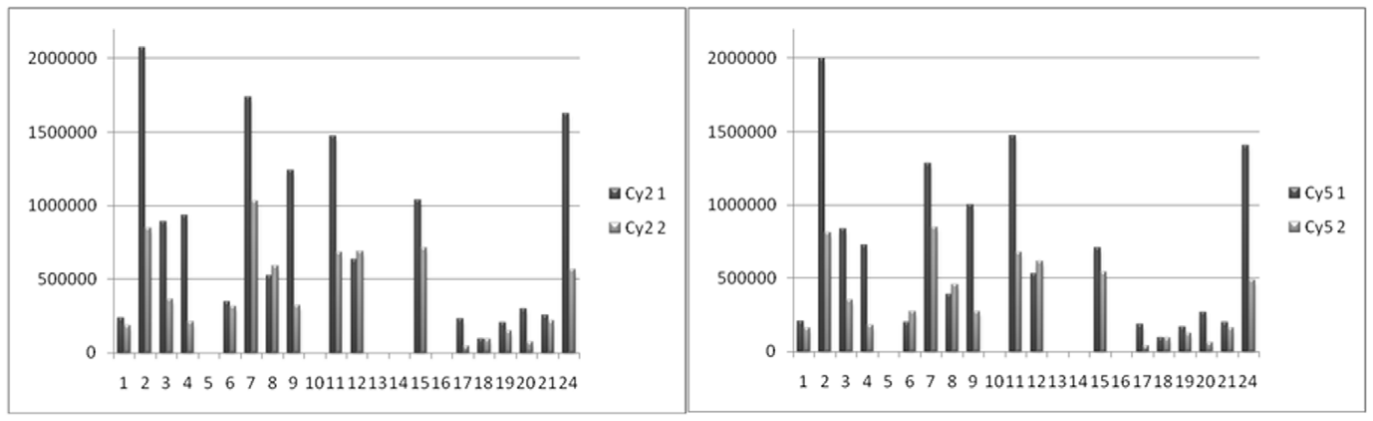
Comparison of spot volumes. Experiments were performed according to the recommended DIGE labelling protocol (10 mM lysine) and increasing the concentration of quencher to 2.5 M (complete quenching). Single experiment: gel 1 (10 mM lysine) – gel 2 (2500 mM lysine), Cy2 image (left) and Cy5 image (right). Data analysis using DIA module.

Having found a lysine concentration which silenced the dye, we proceeded to perform first a single and later replicate experiments comparing both quencher concentrations (gel 1/10 mM, gel 2/2.5 M) in order to determine an estimate for the differences in spot volumes (for protocol see Table S1). Detailed results for the first experiment are given in Table S3. Twenty-five well defined random spots were chosen for analysis across gel 1. Thereby, care was taken not to select proteins which spread over a large area or appeared as spot series. Of these 25 spots, 17 found a corresponding spot in gel 2 by automatic matching. Some spots showed no great change in spot volume within the variations typically observed in two-dimensional gel electrophoresis, but the majority of spots exhibited a much higher spot volume in gel 1 than in gel 2. For 8 spots even more than twice the volume was detected in gel 1 compared to gel 2 (Figure 2; spot 2, 3, 4, 9, 11 17, 20, 24).

**Figure 3:**
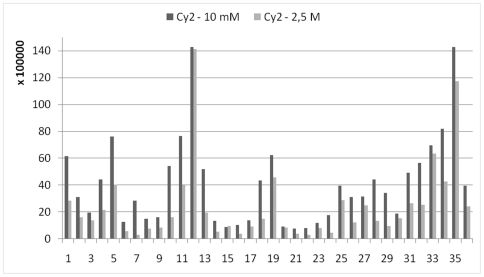
Comparison of spot volumes. Experiments were performed according to the recommended DIGE labelling protocol (10 mM lysine) and increasing the concentration of quencher to 2.5 M (complete quenching). Replicate experiments with 6 gels (gels 1–3 10 mM, gels 4–6 2.5 M. Analysis using BVA module. Shown are the average spot volumes of gels 1–3 *versus* gels 4–6 for Cy2. Unspecific labelling more than doubles the spot volumes in some cases. See Text S2, Text S3.

The goal of the replicate runs was to perform a DIGE-like experiment with three replicate samples, because this is a) the minimum of experiments which should be done for statistical validity and b) the number of experiments often desired by researchers in practice. Three *E. coli* gels were run following recommendations (10 mM lysine [2]) plus three gels where the higher concentration of quencher was used during sample preparation (2.5 M; Text S2, Text S3). Each gel generated a Cy2, Cy3 and Cy5 image, respectively. However, in contrast to DIGE where the Cy2-labelled sample is formed by pooling all samples and used as internal standard, our experiment had to be designed slightly differently to accommodate the fact that Cy2 labelling results in gels 1–3 were expected to differ from those in gels 4–6 due to different quencher concentration. While all gels were run under exact same experimental conditions for best comparison they could, therefore, not be correlated to an internal standard. Spots were matched for all 6 gels and the corresponding spot volumes were compared. All samples, reagents and buffers were freshly prepared. Care was taken to double-check and readjust the required pH values for optimal labelling. Lysine quality would also influence the outcome of the experiment and it was checked by mass spectrometry (Figure S3). Raw volumes of selected spots as well as average spot volumes were evaluated in several ways comparing only few perfectly shaped spots (Text S2) or a larger number of random spots across the gel (Text S3), respectively, using DeCyder DIA and BVA analysis modules. The experimental error became less evident after mathematical data manipulation was performed (Text S2), but even then the results convincingly demonstrated an overabundance of proteins in the gels run with 10 mM lysine. An example is shown in Figure 3 for the average volumes of 36 spots in 3 gels. This image is representative for the two other dyes demonstrating also that individual proteins respond differently to the labelling procedure due to their amino acid sequence, secondary and tertiary structure and possibly due to parameters such as local pH. The introduction of a mathematical factor to correct for the difference in volumes is therefore not feasible. Experiments should be performed using a protocol which ensures that quenching conditions are suitable. The use of 2.5 M instead of 10 mM lysine for quenching serves this purpose in most cases, but a control experiment is advised. Alternatively, a filtration step to physically remove residual dye would also work, but it might also unspecifically remove proteins.

## Materials and Methods

Experiments were performed according to the manufacturer's recommendations [2] as described earlier [1] unless otherwise noted. Detailed information is available in Text S1, Table S1, Table S2, Table S4.

## Supporting Information

Text S1 **Materials and methods.**
(DOC)

Text S2 **Replicated experiments using DIGE protocols (few selected spots).**
(DOC)

Text S3 **Replicated experiments using DIGE protocols (semi-automatic analysis).**
(DOC)

Figure S1 **When labelling is not properly controlled in comparative 2-DE, cross-talk may be observed which results in increased spot volumes.** Thereby, more sample is labelled with the dye assigned for it (e.g., treated sample and Cy3), but the volume increase is also due to the control sample and the pool which both were assigned other dyes (Cy5, Cy2) originally. The cross-labelling occurs when all samples are mixed to be subjected to isoelectric focusing.
(TIF)

Figure S2 **Cy5 images. Left:** Experiment discussed in Figure 1A. 400 pmol Cy5 was first quenched with 10.000 pmol lysine. 50 µg *E. coli* was added into the dye-lysine solution. **Right:** 50 µg *E. coli* was labelled with 40 pmol Cy5 and was then quenched with 100.000 pmol lysine.
(TIF)

Figure S3 **Control of lysine quality.** TSQ-700 (Finnigan) MS/MS spectra of lysine at –15 eV collision energy. Lysine was obtained from Sigma (L-5626) as recommended by GE in the instructions for CyDye DIGE Fluors (minimal dyes) and used as described in Ettan DIGE System User Manual. We have found that storage of lysine stock solution at −20°C does not impair its quality. However, to avoid any issues related to storage, lysine was freshly prepared in this study. In addition, lysine (1 pmol/ml in 0.1% formic acid, 5% acetonitrile) was measured using off-line ion trap mass spectrometry as well as LC-MS with Q-TOF Premier. In both cases the presence of lysine was verified comparing the data to MS measurements with commercial lysine run at the laboratory of Henry M. Fales at the National Institutes of Health in 1998 using TSQ-700 mass spectrometer (poster König/Fales at 48^th^ ASMS conference, Long Beach, CA, 2000). The lysine used here is specified for a purity of >98% and showed the same fragmentation pattern in MS/MS experiments as is demonstrated in this spectrum.
(TIF)

Table S1 **Protocol changes in the experiments discussed in** **Figure 1** **, Figure S2 and Table S3 as compared to the instructions of the manufacturer.**
(DOC)

Table S2 **Buffers and solutions for labelling and 2-DE.**
(DOC)

Table S3 **Volumes of spots matched in the images of gel 1 and gel 2 and their differences.** Each gel carried three samples labelled with Cy2, Cy3, or Cy5, respectively, but the amount of quencher was different (Table S1).
(DOC)

Table S4 **Scan parameters for the six gels of** ***E. coli*** **proteins.**
(DOC)
