# Supplementary material for: Impact of Quenching Failure of Cy Dyes in Differential Gel Electrophoresis
Source: PLoS One. 2011 Mar 30;6(3):e18098. doi: 10.1371/journal.pone.0018098 (PMC3068157; doi:10.1371/journal.pone.0018098)
Supplement: Text S1 — Materials and methods. (DOC) [file pone.0018098.s001.doc]

**Text S1: Materials and methods**

All components for DIGE such as devices, dyes and software (Immobiline DryStrips pH 3-10 L, DryStrip Reswelling Tray, Ettan^TM^ IPGphor II^TM^ including the Cup Loading Manifold, Ettan^TM^ DALTsix and gel caster, Multi Temp III, Typhoon 9400, Image Quant, Decyder 2D 6.5) were obtained from GE Healthcare except acrylamide, N,N’-methylenebisacrylamide (Bio-Rad). Other chemicals were purchased from Sigma-Aldrich including *Escherichia coli* EC11303.

*E. coli* lysate was extracted from 25.77 mg into 1 ml lysis buffer (30 mM Tris, 8 M urea, 4 % (w/v) CHAPS, pH 8.79) for 30 min at room temperature under vortexing (VIBRAX VXR 1500/min). Then the sample was centrifuged for 10 min (12.000 xg, 4 °C). The clear supernatant was removed into a new tube. The protein concentration of *E. coli* was determined using a spectrophotometer (Ultrospec 2000, Pharmacia Biotech) at 590 nm. The pH values of the sample and buffer were determined with a pH meter (TitroLine easy and Orion 8220 electrode). The concentration and pH value were checked regularly.

Sample labelling and gel electrophoresis were carried out according to the manufacturer´s instructions (Ettan DIGE User Manual, Edition AA 2003, Amersham/GE Healthcare) unless otherwise specified. For isoelectric focusing 24 cm DryStrips pH 3-10 were used. The second dimension was run at 20 °C on 12.5 % SDS-PAGE gels. The gels were scanned directly or after being stored at 4 °C overnight in the dark with Typhoon 9400 variable imager using the blue laser (488 nm) for Cy2 (emission filter 520 BP 40), the green laser (532 nm) for Cy3 (580 BP 30) and the red laser (633 nm) for Cy5 (670 BP 30). Image analysis and spot detection were performed with DeCyder software. In the DIA module, the estimated number of spots was set to 2500.
