# Supplementary material for: Impact of Quenching Failure of Cy Dyes in Differential Gel Electrophoresis
Source: PLoS One. 2011 Mar 30;6(3):e18098. doi: 10.1371/journal.pone.0018098 (PMC3068157; doi:10.1371/journal.pone.0018098)

**Text S2: Replicated experiments using DIGE protocols (few selected spots)**

The general goal was to perform a DIGE experiment with three replicates, because this is a) the minimum of experiments, which should be run for statistical validity and b) the number of experiments often desired by researchers in practice. In our case, 3 gels were run according to the recommended labelling procedure using 10 mM lysine (gel 1-3) and 3 gels using the corrected protocol with 2.5 M lysine (gel 4-6). Each gel produced a Cy2-, Cy3- and Cy5-image, respectively. However, in contrast to the typical DIGE experiment where the Cy2 sample is used as internal standard formed by a pool of all samples in the experiment, our experiment had to be designed slightly differently to accommodate the fact that Cy2 labelling in gels 1-3 is expected to differ from that in gels 4-6. While all gels were run under exact same experimental conditions for best comparison (with the exception of quenching) they could not be matched to a single internal standard. Spots were matched for all 6 gels and the corresponding spot volumes were compared.

As before, *E. coli* lysate was used as test proteome. All samples, reagents and buffers were freshly prepared. 2-DE was performed as discussed above. Care was taken to double-check and readjust the required (and recommended) pH values for optimal labelling. In all experiments 50 µg protein was labelled with 400 pmol dye; only the concentration of the quencher differed between gel group 1-3 and 4-6. Typhoon scan parameters for all gels are shown in Table S4. PMT values had to be adjusted for optimal scanning results as is a typical situation in DIGE experiments.

Software DeCyder was employed for analysis, initially using DIA module. Spot detection was performed setting the expected spot number to 2500. Spot matching was automatically done and manually corrected. Seven well defined spots of Gaussian appearance were chosen for volume comparison thereby avoiding any spots with shoulders, smearing or other properties which would influence volume determination. Both raw volume and normalized volume (raw volume / average volume) were calculated. Data are shown below.


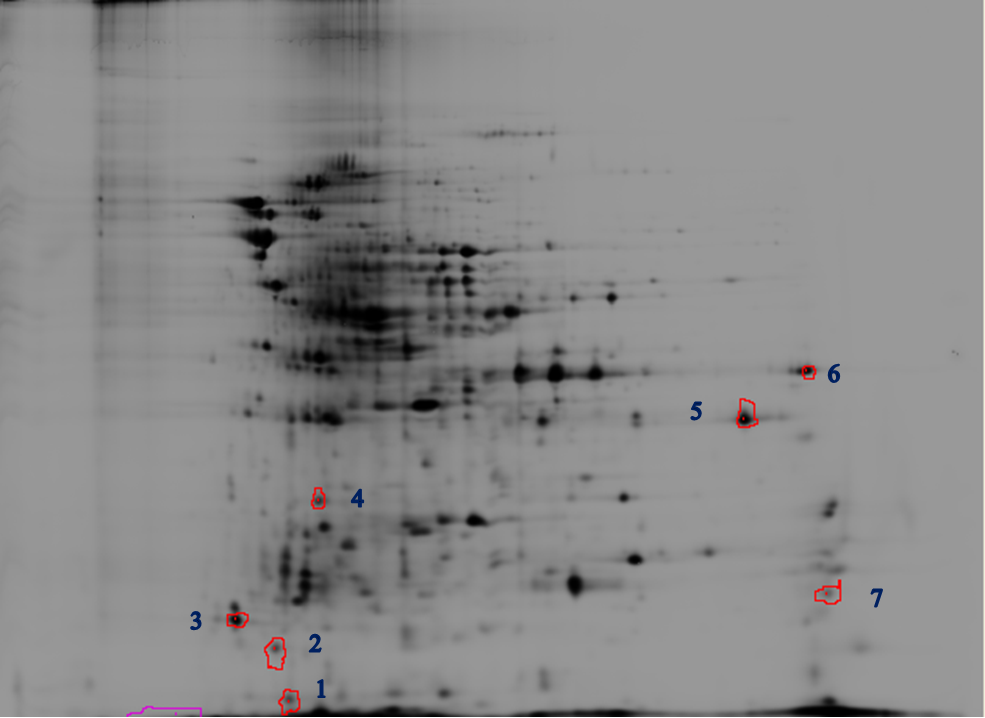
Location of selected spots in 6 gels and schematics of their volumes (sum of raw volumes in gels 1-3 and gels 4-6, respectively, divided by 3, and of normalized volumes).


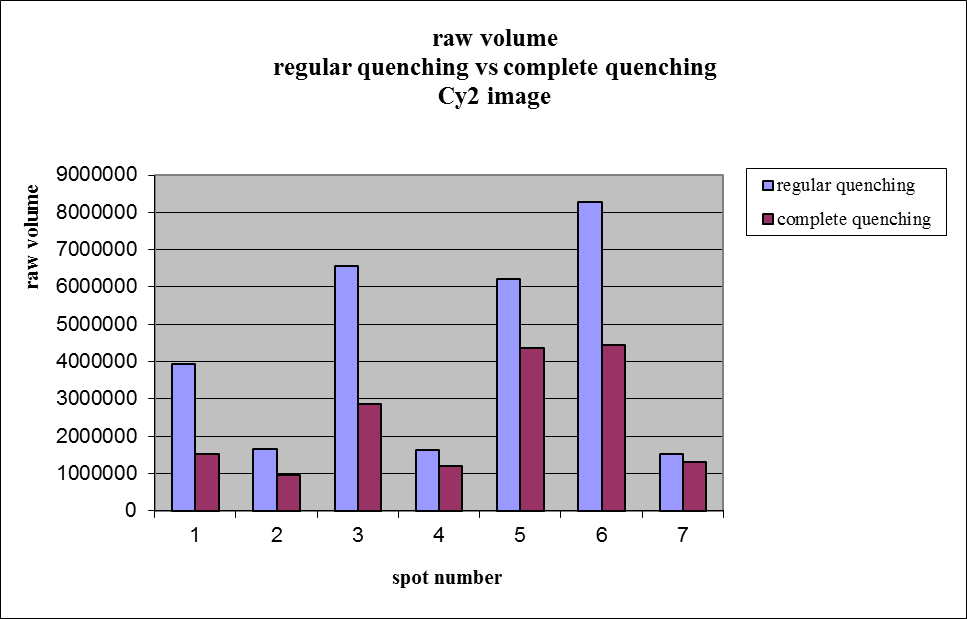


*
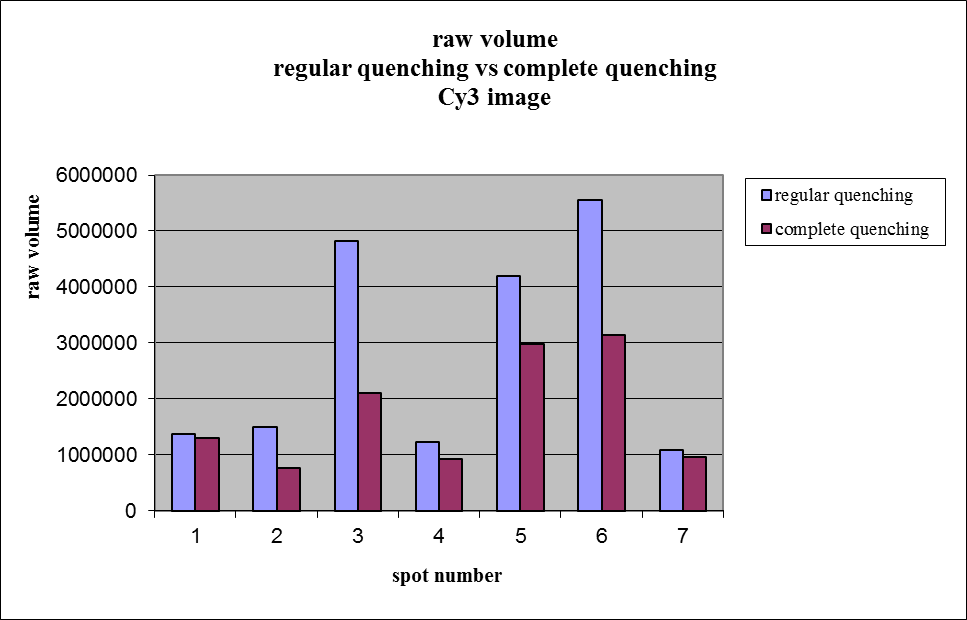
*


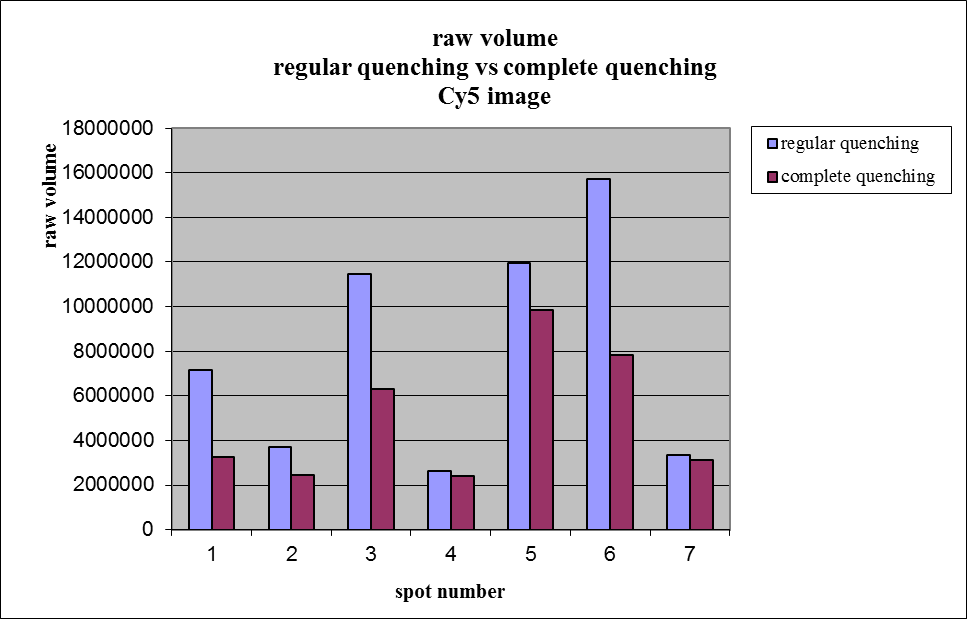


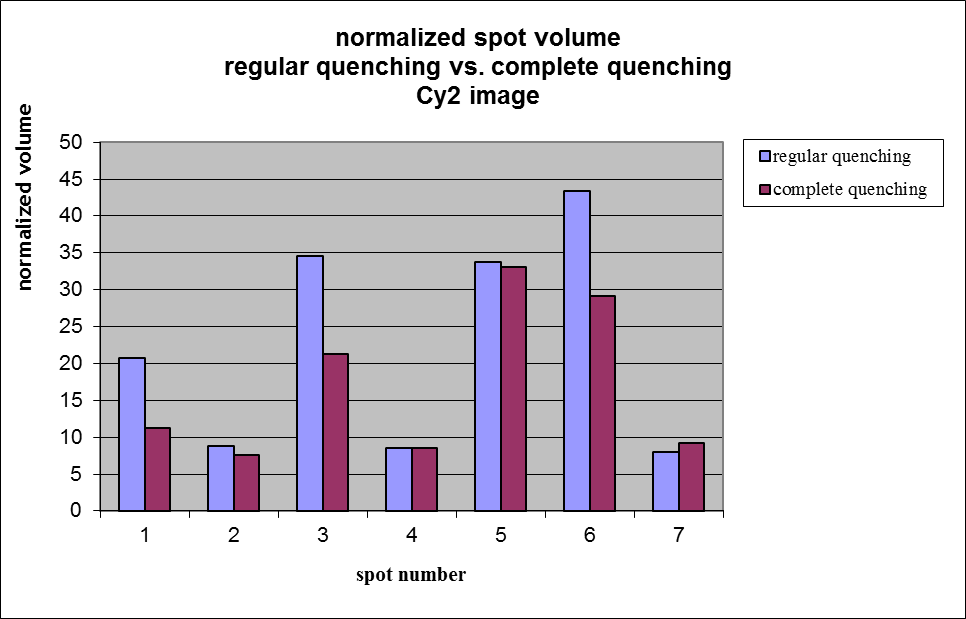


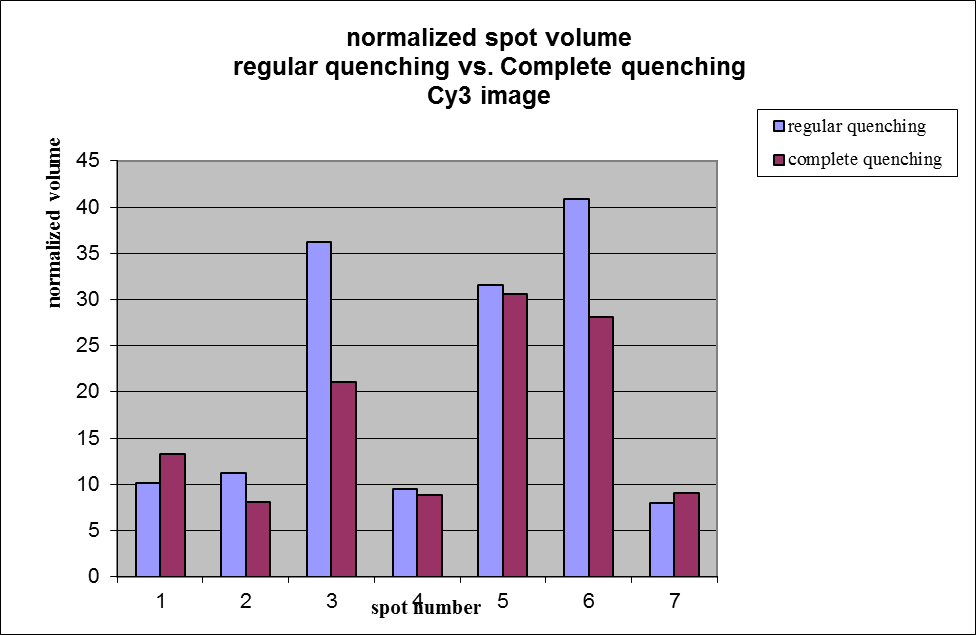


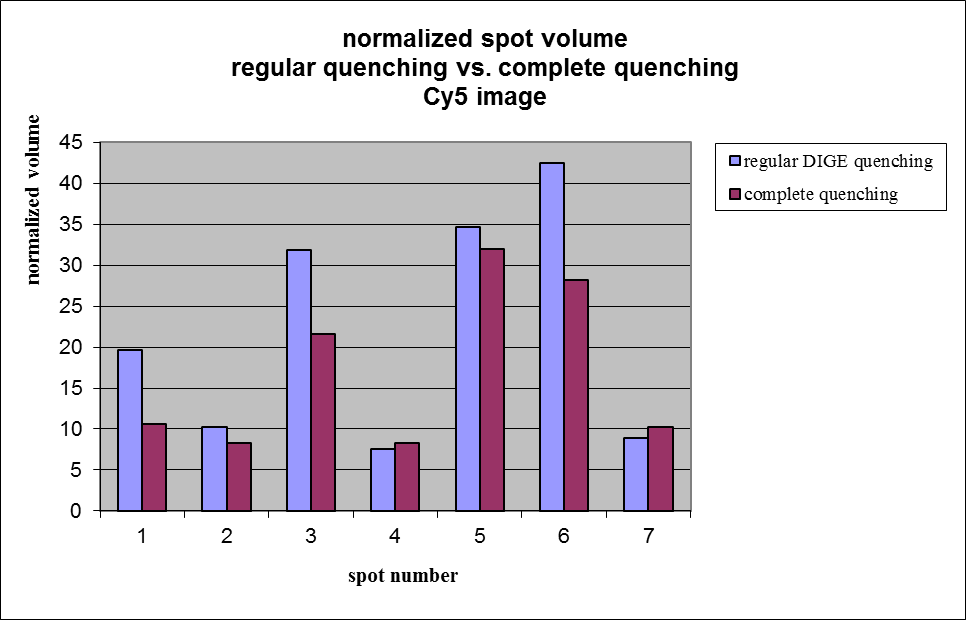

Supplement: Text S2 — Replicated experiments using DIGE protocols (few selected spots). (DOC) [file pone.0018098.s002.doc]
