# Supplementary material for: Impact of Quenching Failure of Cy Dyes in Differential Gel Electrophoresis
Source: PLoS One. 2011 Mar 30;6(3):e18098. doi: 10.1371/journal.pone.0018098 (PMC3068157; doi:10.1371/journal.pone.0018098)

**Text S3: Replicated experiments using DIGE protocols (semi-automatic analysis)**

In order to test a situation closer to real experiments, a larger number of spots were selected in DyCyder BVA module and raw volumes were compared.


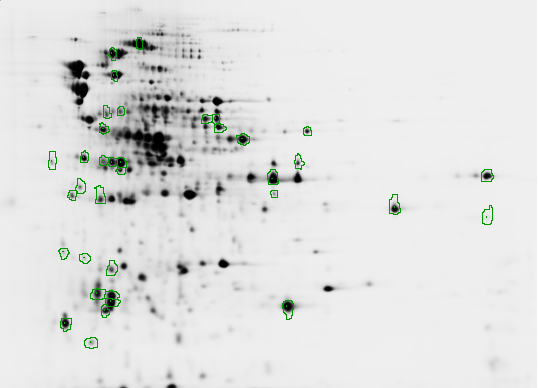
BVA analysis of 6 *E. coli* gels. 36 spots were chosen for volume comparison.

Data were visualized in different graphical formats below.


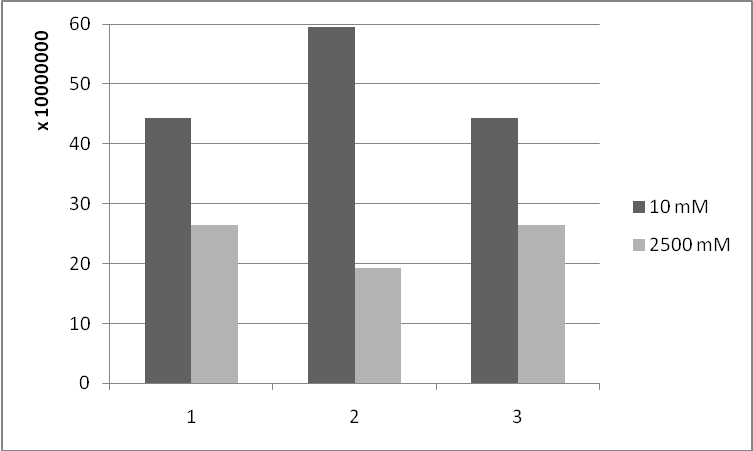
Average spot volumes from left to right: Cy2, Cy3, Cy5.

Following diagrams: Average spot volumes in gels 1-3 *versus* gels 4-6.


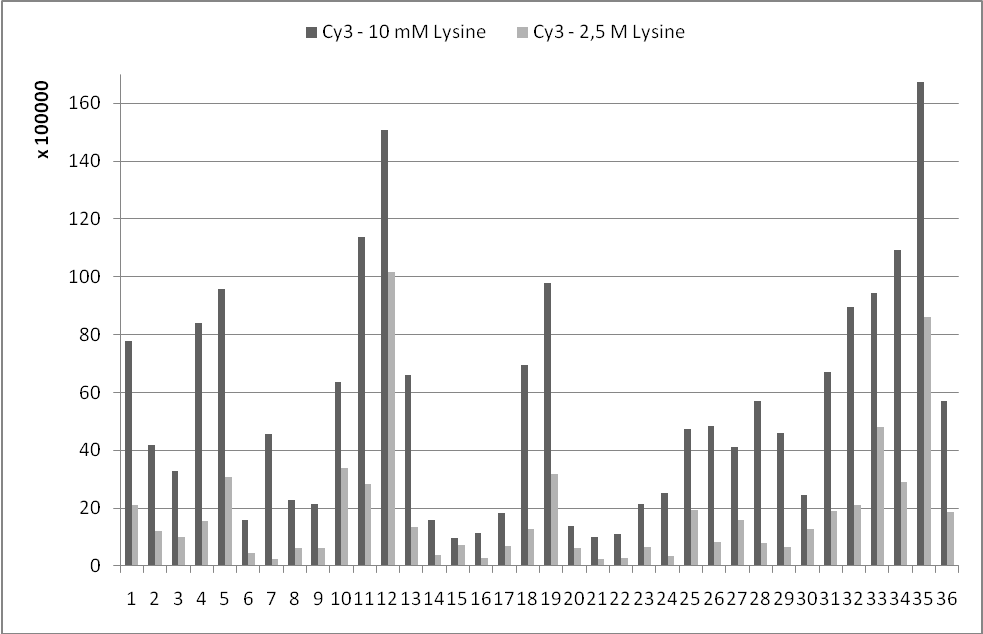


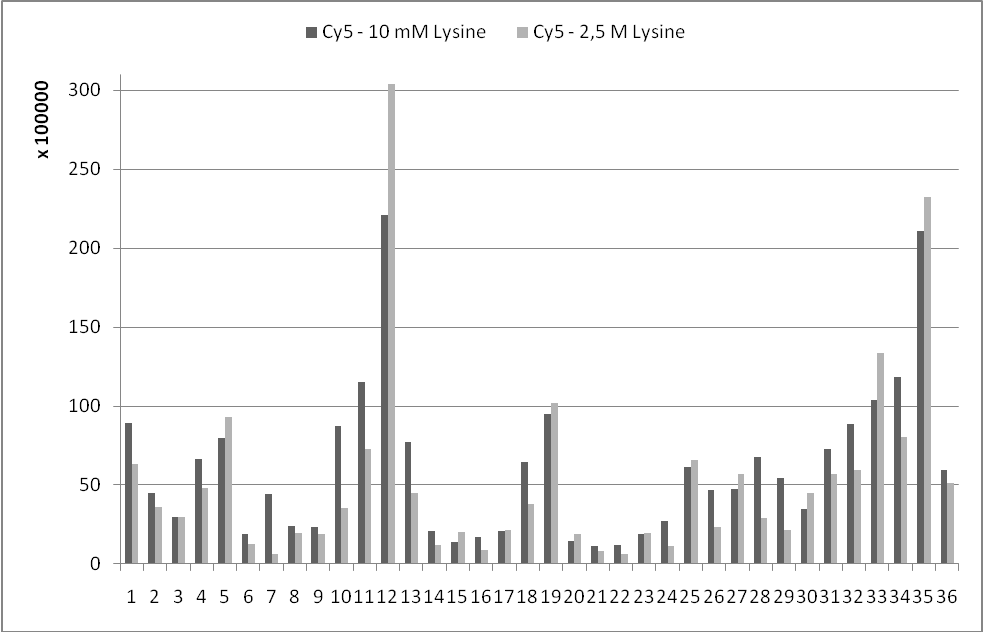

Supplement: Text S3 — Replicated experiments using DIGE protocols (semi-automatic analysis). (DOC) [file pone.0018098.s003.doc]
