# Supplementary figures and images for: Impact of Quenching Failure of Cy Dyes in Differential Gel Electrophoresis
Source: PLoS One. 2011 Mar 30;6(3):e18098. doi: 10.1371/journal.pone.0018098 (PMC3068157; doi:10.1371/journal.pone.0018098)

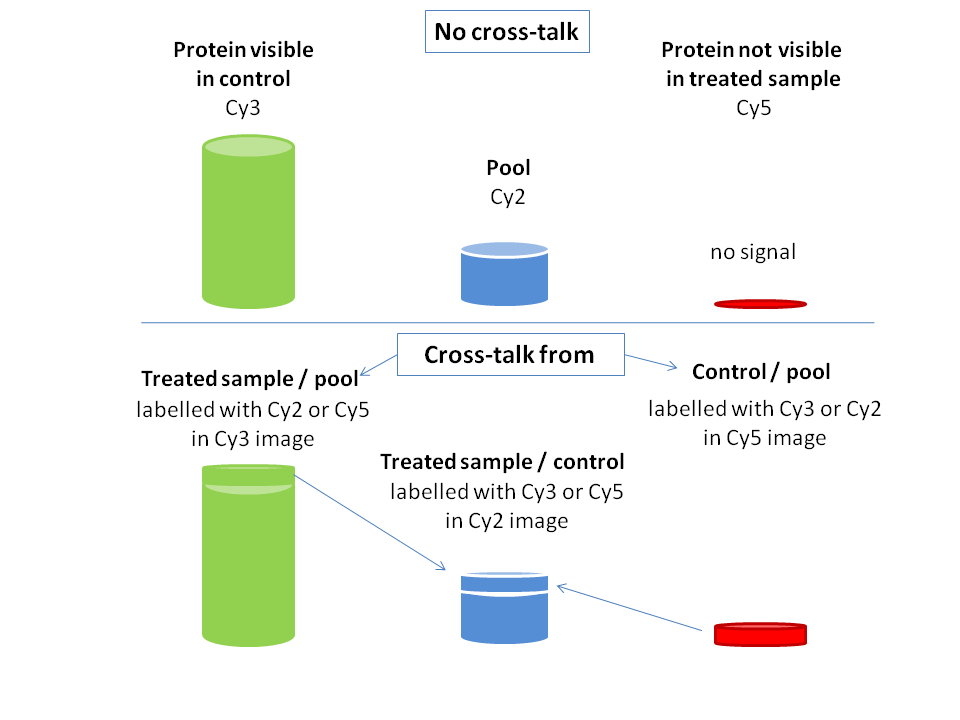

Supplement: Figure S1 — When labelling is not properly controlled in comparative 2-DE, cross-talk may be observed which results in increased spot volumes. Thereby, more sample is labelled with the dye assigned for it (e.g., treated sample and Cy3), but the volume increase is also due to the control sample and the pool which both were assigned other dyes (Cy5, Cy2) originally. The cross-labelling occurs when all samples are mixed to be subjected to isoelectric focusing. (TIF) [file pone.0018098.s004.tif]

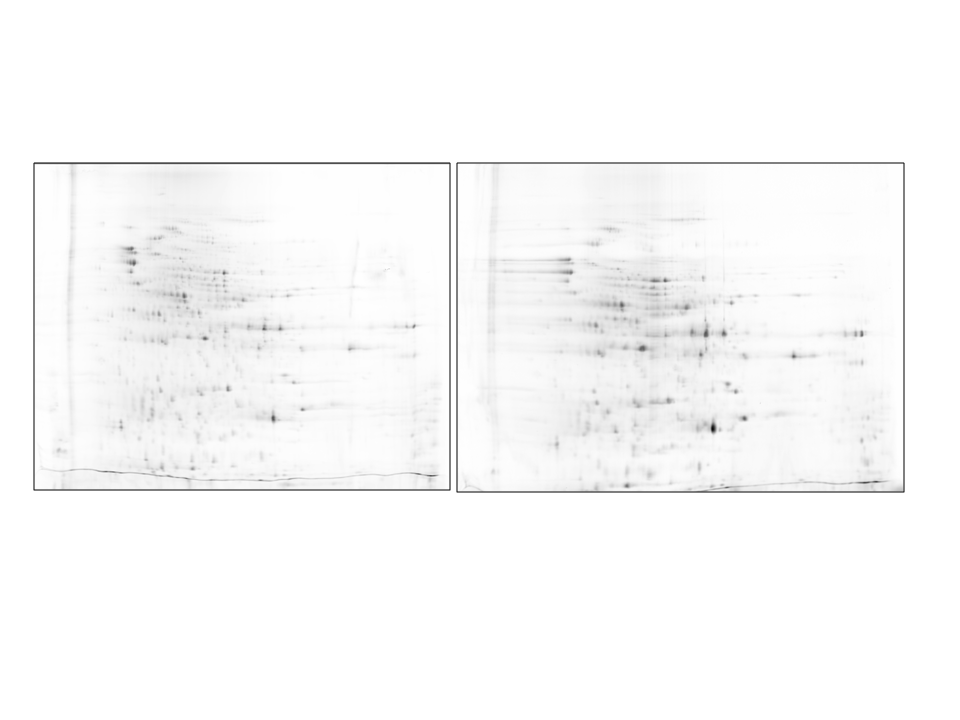

Supplement: Figure S2 — Cy5 images. Left: Experiment discussed in Figure 1A. 400 pmol Cy5 was first quenched with 10.000 pmol lysine. 50 µg E. coli was added into the dye-lysine solution. Right: 50 µg E. coli was labelled with 40 pmol Cy5 and was then quenched with 100.000 pmol lysine. (TIF) [file pone.0018098.s005.tif]

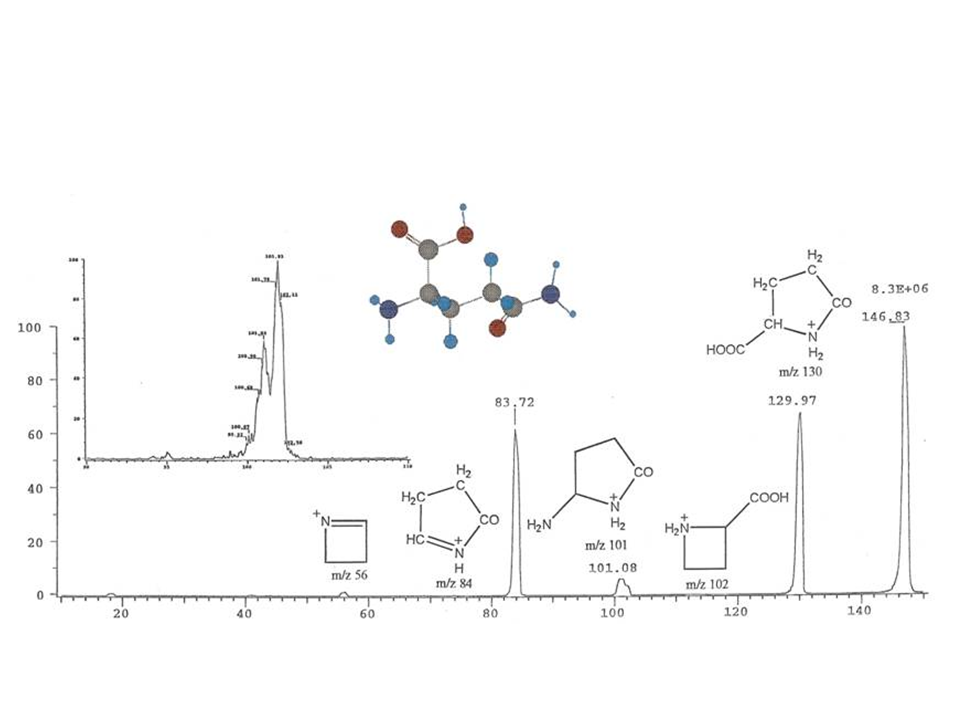

Supplement: Figure S3 — Control of lysine quality. TSQ-700 (Finnigan) MS/MS spectra of lysine at –15 eV collision energy. Lysine was obtained from Sigma (L-5626) as recommended by GE in the instructions for CyDye DIGE Fluors (minimal dyes) and used as described in Ettan DIGE System User Manual. We have found that storage of lysine stock solution at −20°C does not impair its quality. However, to avoid any issues related to storage, lysine was freshly prepared in this study. In addition, lysine (1 pmol/ml in 0.1% formic acid, 5% acetonitrile) was measured using off-line ion trap mass spectrometry as well as LC-MS with Q-TOF Premier. In both cases the presence of lysine was verified comparing the data to MS measurements with commercial lysine run at the laboratory of Henry M. Fales at the National Institutes of Health in 1998 using TSQ-700 mass spectrometer (poster König/Fales at 48th ASMS conference, Long Beach, CA, 2000). The lysine used here is specified for a purity of >98% and showed the same fragmentation pattern in MS/MS experiments as is demonstrated in this spectrum. (TIF) [file pone.0018098.s006.tif]
