# Supplementary material for: Impact of Quenching Failure of Cy Dyes in Differential Gel Electrophoresis
Source: PLoS One. 2011 Mar 30;6(3):e18098. doi: 10.1371/journal.pone.0018098 (PMC3068157; doi:10.1371/journal.pone.0018098)
Supplement: Table S1 — Protocol changes in the experiments discussed in Figure 1 , Figure S2 and Table S3 as compared to the instructions of the manufacturer. (DOC) [file pone.0018098.s007.doc]

**Table S1:** Protocol changes in the experiments discussed in Figure 1, Figure S2 and Table S3 as compared to the instructions of the manufacturer. After step 3 all samples were diluted with 2X sample buffer. For buffers and solutions see Table S2.

|  | **Step 1** | **Step 2** | **Step 3** |
| --- | --- | --- | --- |
| **DIGE [3]** | labelling:  50 µg *E. coli*  400 pmol dye | quenching:  1 µl of 10 mM L-lysine  (10.000 pmol) | sample pool |
| **Figure 1A**  **Cy2 image** | quenching:  1 µl of 10 mM L-lysine  (10.000 pmol)  9 µl lysis buffer  400 pmol dye | add:  50 µg *E. coli*  7.28 µl  30 min, on ice in the dark | 1 sample  per gel |
| **Figure 1B**  **Cy2 image** | quenching:  1 µl of 2.5 M L-lysine  (2.500.000 pmol)  9 µl lysis buffer  400 pmol dye | add:  50 µg *E. coli*  9.52 µl  30 min, on ice in the dark | 1 sample  per gel |
| **Figure S2**  **Cy5 image**  **right** | labelling:  50 µg *E. coli*  40 pmol dye | quenching:  1 µl of 100 mM L-lysine  (100.000 pmol) | sample pool |
| **Table S3** | labelling:  50 µg *E. coli*  400 pmol dye | quenching:  gel 1: 1 µl of 10 mM L-lysine  (10.000 pmol)  gel 2: 1 µl of 2.5 M L-lysine  (2.500.000 pmol) | sample pool |
