# Supplementary material for: Impact of Quenching Failure of Cy Dyes in Differential Gel Electrophoresis
Source: PLoS One. 2011 Mar 30;6(3):e18098. doi: 10.1371/journal.pone.0018098 (PMC3068157; doi:10.1371/journal.pone.0018098)
Supplement: Table S2 — Buffers and solutions for labelling and 2-DE. (DOC) [file pone.0018098.s008.doc]

**Table S2:** Buffers and solutions for labelling and 2-DE.

| **Step** | **Operation** | **T / °C** | **pH** | **Buffer** |
| --- | --- | --- | --- | --- |
| 1 | sample preparation | 4 °C | 8.5 | lysis buffer |
| 2 | preparation of CyDye stock and working solution | - | - | DMF |
| 3 | labelling | 4 °C | 8.5 | lysis buffer, DMF |
| 4 | quenching with 1 µl 10 mM lysine or 2.5 M lysine | 4 °C | - | lysis buffer, DMF |
| 5 | pooling labelled samples | 4 °C | - | lysis buffer, DMF |
| 6 | diluting sample 1:2 with sample buffer | 4 °C | - | lysis buffer, DMF, sample buffer |
| 7 | 1st dimension | 20 °C | - | lysis buffer, DMF, sample buffer, rehydration buffer, mineral oil |
| 8 | equilibration | RT | - | equilibration buffer 1  equilibration buffer 2 |
| 9 | 2nd dimension | 20 °C | - | 2X running buffer |

*Lysis buffer:* 30 mM Tris, 8 M urea, 4 % CHAPS, pH adjusted to 8.5 at 4 °C with 0.1 M HCl

*Sample buffer*: 8 M urea, 4 % (w/v) CHAPS, 2 % (v/v) Pharmalyte^TM^ pH 3-10, 2 % (w/v) DTT

*Rehydration buffer:* 8 M urea, 4 % (w/v) CHAPS, 1 % (v/v) Pharmalyte^TM^ pH 3-10, 0.2 % (w/v) DTT, 0.004 % (w/v) bromophenol blue

*Equilibration buffer 1:* 100 mM Tris buffer adjusted with HCl to pH 8, 6 M urea, 30 % (v/v) glycerol (87 % v/v), 2 % SDS (w/v), 1 % (w/v) DTT

*Equilibration buffer 2:* 100 mM Tris buffer adjusted with HCl to pH 8, 6 M urea, 30 % (v/v) glycerol (87 % v/v), 2 % SDS (w/v), 2.5 % (w/v) iodoacetamide

*10X running buffer:* 0.25 M Tris; 1.92 M glycine, 2 % (w/v) SDS

*2X running buffer:* 1:5 (v/v) dilution from 10X running buffer
