# Supplementary material for: Impact of Quenching Failure of Cy Dyes in Differential Gel Electrophoresis
Source: PLoS One. 2011 Mar 30;6(3):e18098. doi: 10.1371/journal.pone.0018098 (PMC3068157; doi:10.1371/journal.pone.0018098)

**Table S3:** Volumes of spots matched in the images of gel 1 and gel 2 (see main text; for spot location see image, next page) and their differences. Each gel carried three samples labelled with Cy2, Cy3, or Cy5, respectively, but the amount of quencher was different (Table S1). Both gels were scanned at PMT 500. The images were analysed with DeCyder 2D software. Spot detection in DIA was set to 2500 and spot matching in BVA was run automatically. 25 well defined spots were selected on gel 1. 17 of them could be assigned to a spot on gel 2. The raw spot volumes are listed below in tabular and graphical form (see also Figure 2).

|  | **Cy2 1** | **Cy2 2** | **1-2** | **Cy3 1** | **Cy3 2** | **1-2** | **Cy5 1** | **Cy5 2** | **1-2** |
| --- | --- | --- | --- | --- | --- | --- | --- | --- | --- |
| **1** | 234341 | 185260 | 49081 | 164244 | 127710 | 36534 | 205257 | 161630 | 43627 |
| **2** | 2069596 | 845742 | 1223854 | 1542981 | 571396 | 971585 | 2001135 | 811983 | 1189152 |
| **3** | 887447 | 365990 | 521457 | 612505 | 229157 | 383348 | 836960 | 349293 | 487667 |
| **4** | 933189 | 211245 | 721944 | 641951 | 142306 | 499645 | 725792 | 180054 | 545738 |
| **6** | 346852 | 316542 | 30310 | 193647 | 184953 | 8694 | 196072 | 273449 | -77377 |
| **7** | 1735646 | 1028722 | 706924 | 1279557 | 718414 | 561143 | 1279557 | 847822 | 431735 |
| **8** | 522437 | 590144 | -67707 | 387501 | 419104 | -31603 | 387944 | 456492 | -68548 |
| **9** | 1236741 | 321701 | 915040 | 776193 | 200216 | 575977 | 999457 | 272079 | 727378 |
| **11** | 1469020 | 680295 | 788725 | 1132002 | 508772 | 623230 | 1469020 | 674990 | 794030 |
| **12** | 632775 | 686917 | -54142 | 451935 | 505594 | -53659 | 527245 | 613949 | -86704 |
| **15** | 1038952 | 711635 | 327317 | 743719 | 517160 | 226559 | 703896 | 538126 | 165770 |
| **17** | 227223 | 46959 | 180264 | 156163 | 28386 | 127777 | 184305 | 40411 | 143894 |
| **18** | 95872 | 92299 | 3573 | 73417 | 57394 | 16023 | 95872 | 92299 | 3573 |
| **19** | 206007 | 148974 | 57033 | 144089 | 89270 | 54819 | 167896 | 121492 | 46404 |
| **20** | 297545 | 72559 | 224986 | 199596 | 42560 | 157036 | 267916 | 60439 | 207477 |
| **21** | 252005 | 217054 | 34951 | 173320 | 125482 | 47838 | 197472 | 162717 | 34755 |
| **24** | 1622022 | 564886 | 1057136 | 1199096 | 375028 | 824068 | 1402268 | 488148 | 914120 |
| **Σ** |  |  | **6720746** |  |  | **5029014** |  |  | **5502691** |


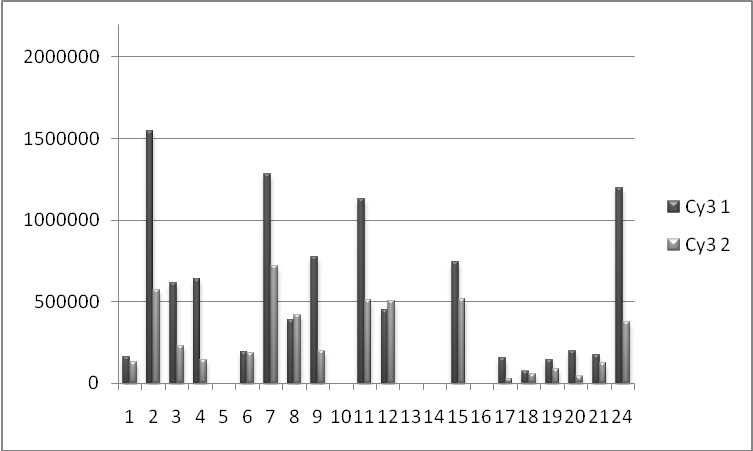

Supplement: Table S3 — Volumes of spots matched in the images of gel 1 and gel 2 and their differences. Each gel carried three samples labelled with Cy2, Cy3, or Cy5, respectively, but the amount of quencher was different (Table S1). (DOC) [file pone.0018098.s009.doc]
