# Supplementary material for: Impact of Quenching Failure of Cy Dyes in Differential Gel Electrophoresis
Source: PLoS One. 2011 Mar 30;6(3):e18098. doi: 10.1371/journal.pone.0018098 (PMC3068157; doi:10.1371/journal.pone.0018098)
Supplement: Table S4 — Scan parameters for the six gels of E. coli proteins. (DOC) [file pone.0018098.s010.doc]

**Table S4:** Scan parameters for the six gels of *E. coli* proteins.

| **Gel** | **Sample** | **PMT** | **High** | **Low** |
| --- | --- | --- | --- | --- |
| 1 | Ecoli-Cy3 | 505 | 99829,84 | 3,11 |
|  | Ecoli-Cy5 | 530 | 99969,35 | 2,05 |
|  | Ecoli-Cy2 | 505 | 99559,83 | 13,3 |
| 2 | Ecoli-Cy3 | 500 | 98864.97 | 3,28 |
|  | Ecoli-Cy5 | 510 | 92898,83 | 0,98 |
|  | Ecoli-Cy2 | 505 | 96476,64 | 13,05 |
| 3 | Ecoli-Cy3 | 500 | 99012,21 | 13,05 |
|  | Ecoli-Cy5 | 530 | 99934,47 | 2,17 |
|  | Ecoli-Cy2 | 505 | 99012,21 | 13,05 |
| 4 | Ecoli-Cy3 | 500 | 99485,86 | 3,70 |
|  | Ecoli-Cy5 | 505 | 92344,73 | 1,05 |
|  | Ecoli-Cy2 | 500 | 99172,86 | 12,66 |
| 5 | Ecoli-Cy3 | 510 | 99799,34 | 4,42 |
|  | Ecoli-Cy5 | 545 | 99973,71 | 3,02 |
|  | Ecoli-Cy2 | 510 | 99242,38 | 13,92 |
| 6 | Ecoli-Cy3 | 500 | 99577,24 | 3,36 |
|  | Ecoli-Cy5 | 540 | 99873,43 | 2,94 |
|  | Ecoli-Cy2 | 500 | 99485,86 | 11,14 |
